# Assessment of the Mechanical Properties and Fragment Characteristics of a 3D-Printed Forearm Orthosis

**DOI:** 10.3390/polym16233349

**Published:** 2024-11-29

**Authors:** Mislav Majdak, Slavica Bogović, Maja Somogyi Škoc, Iva Rezić Meštrović

**Affiliations:** 1 Department of Applied Chemistry, University of Zagreb Faculty of Textile Technology, 10000 Zagreb, Croatia; iva.rezic@ttf.unizg.hr; 2 Department of Clothing Technology, University of Zagreb Faculty of Textile Technology, 10000 Zagreb, Croatia; slavica.bogovic@ttf.unizg.hr; 3 Department of Materials, Fibres and Textile Testing, University of Zagreb Faculty of Textile Technology, 10000 Zagreb, Croatia; maja.somogyi@ttf.unizg.hr

**Keywords:** orthosis, 3D printing, 3D modelling, 3D scanning, mechanical properties, microscopic characterization, medical textiles

## Abstract

Distal radius fractures (DRF) are one of the most prevalent injuries a person may sustain. The current treatment of DRF involves the use of casts made from Plaster of Paris or fiberglass. The application of these materials is a serious endeavor that influences their intended use, and should be conducted by specially trained personnel. In this research, with the use of the full-body 3D scanner Vitus Smart, 3D modelling software Rhinoceros 3D, and 3D printer Creality CR-10 max, an easy, yet effective workflow of orthosis fabrication was developed. Furthermore, samples that represent segments of the orthosis were subjected to static loading. Lastly, fragments that occurred due to excessive force were characterized with the use of a digital microscope. It was observed that with the implementation of the designed workflow, a faster 3D printing process was present. Samples subjected to mechanical loading had values that exceeded those of conventional Plaster of Paris; the minimum recorded value was 681 N, while the highest was 914 N. Microscopic characterization enabled a clear insight into the occurrence of fragments, as well as their potential risk. Therefore, in this research, an insight into different stages of fabrication, characterization of undesirable events, as well as the risks they may pose were presented.

## 1. Introduction

Distal radius fractures (DRF) are one of the most common injuries a person may sustain during their lifetime, especially in their youth and senior years. According to the recent studies, it is estimated that DRF make up 17.8% and 17.5% of all fractures sustained by these two demographic groups, respectively, while the cause of said fractures is most commonly a fall on an outstretched arm while trying to break the fall [1,2,3]. As for the reasons why these two groups are mostly affected, an increase in physical activity among children and osteoporosis among the elderly, paired with the unfortunate rise in obesity, which may lead to the decrease in bone mass, have been identified as the main culprits [4,5,6,7].

Currently, any type of fracture is either treated by the use of conservative or surgical methods of treatment. In the case of DRF, this either involves the use of a short-arm cast in cases of conservative treatment, or devices such as Volar locking plates in cases of surgical treatment [8,9,10]. Even though in recent years surgical treatment has seen a greater increase in use, the conservative methods of treatment, such as various casts, are still widely used, since there is no definitive evidence that one is superior to another [11,12].

For the last couple of centuries, casts were primarily made of Plaster of Paris textile composite materials, but today, they are slowly being replaced by casts made from polymer materials, such as fiberglass; the reasons for this being better stability when in contact with water, smaller weight, and better durability. Regardless of their material composition, casts are applied in an established procedure that involves: (i) inspection of an affected limb for any swelling or possible neurovascular problems; (ii) application of medical textiles; a stockinette and cotton padding; and (iii) application of soaked casting textile bandages in a figure of eight pattern, with an immediate rubbing of said bandages for an increased cohesion. Afterwards, the cast is left to set [13,14]. It is generally assumed that the setting process only takes few minutes, while the drying process takes 36 to 72 h. As the cast dries, its mechanical properties increase, and only after it fully dries can the cast bear a complete load. In addition to the drying time, number of layers as well as the successful application process heavily influence the mechanical properties of the cast [15,16,17]. The application process is a serious and crucial step that heavily relies on the knowledge and adequate training of the personnel in charge; otherwise, serious problems regarding patients’ wellbeing may occur. These are most commonly problems such as compartment syndrome and venous congestion, which may occur if potential swelling is overlooked and the bandages are applied too tight. On the other hand, even though severe skin burns caused by the sudden generation of heat during the soaking and layering of plaster bandages rarely occur, they may still pose a serious threat [14,18,19]. Other than the aforementioned problems, conventional casts lack proper ventilation, i.e., breathability, which in turn enables conditions for the spread of bacteria, which leads to the spread of odor [20]. Therefore, in recent years, advanced orthotics are being researched. These devices are known for properties that conventional ones lack, more precisely, the individualistic design and even lower weight (3D-printed cast—318 g; fiberglass cast—325 g; Plaster of Paris cast—457 g) [21,22,23]. According to the ISO 22523:2006, external orthoses are medical devices intended to assist neuro-muscular and skeletal systems. These devices can be constructed from single or multiple pieces, and most importantly, they have to abide by the ergonomic principles, meaning that the end user’s needs have to be taken into great consideration [24]. To successfully achieve this, 3D scanning and 3D printing can be utilized. With the use of 3D scanners, accurate anthropometric measurements can be obtained in a safe and relatively fast manner [25]. Furthermore, with the use of various 3D printing technologies, these individualized objects can be reliably fabricated, and in comparison to more traditional production methods that would rely on expensive molds, in a more cost and time effective manner [26,27]. Even though there are various 3D printing technologies present today, the most noteworthy, especially in the field of medical device fabrication, is fused deposition modelling (FDM) [28,29,30]. The method in question relies exclusively on the use of polymers, such as poly lactic acid (PLA), acrylonitrile butadiene styrene (ABS), polyamide (PA), polyethylene terephthalate (PET), and poly-ε-caprolactone (PCL) [31]. Out of these polymers, PLA is the most commonly used, as it makes up approximately 86% of all build materials used by FDM printers [28]. PLA is an aliphatic polyester derived from lactic acid, whose most noteworthy properties are biocompatibility and biodegradability [32]. Moreover, one of the main reasons for a widespread use of PLA is the fact that lactic acid is produced from an abundant and readily available source, starch, which is mostly obtained from corn or potatoes [33]. Regarding its mechanical properties, in spite of the inherent brittleness, tensile strength (60–70 MPa) and Young’s modulus (2–4 GPa) of PLA are larger than that of PCL [32,34,35]. Unfortunately, due to its low glass transition temperature, between 50 and 70 °C, mechanical properties of PLA change with the increase in the temperature, although a more dramatic change occurs around the aforementioned temperature range [36]. Furthermore, in comparison to the other established polymer, ABS, PLA has a lower volatile organic compound content and lower emission of particles during the printing process, which makes it safer for indoor printing [37]. For the purposes of this research, PLA was chosen as a build material not only because of its aforementioned properties, but also due to its affordable price and ease of printing, which many thermoplastic polymers that were investigated for use in orthopedics, shown in Table 1, lack.

In previous research that dealt with the fabrication and characterization of orthotic devices, a great deal of effort was placed on the investigation of various build materials and their properties, especially mechanical properties, and optimization of said materials in an effort to produce or indicate a production of the best possible orthotic devices [21,22,23]. Furthermore, in some of the papers, the 3D modelling process comes off as convoluted due to various software used in this process [44,45,46]. Therefore, in this paper, a great deal of focus will be placed on expanding of the 3D modelling process, started by Li and Tanaka [47], with the emphasis on the ease of modelling by focusing on a simple geometry. Furthermore, mechanical properties, more precisely, the maximum loading capacity of test samples that represent the segments of the finished 3D model, will be investigated. By doing so, optimum infill density and printing time of the final model will be decided. Finally, fragments that occur after the break will be characterized with a digital microscope. This characterization is intended to visualize and describe the occurrence of fragments and jagged pieces that may pose a piercing risk in case of the orthosis failure. By doing so, a contribution to the understanding of the safety aspects that have not been addressed in previous research [21,22,44,45] will be given.

## 2. Materials and Methods

**Figure 1:**
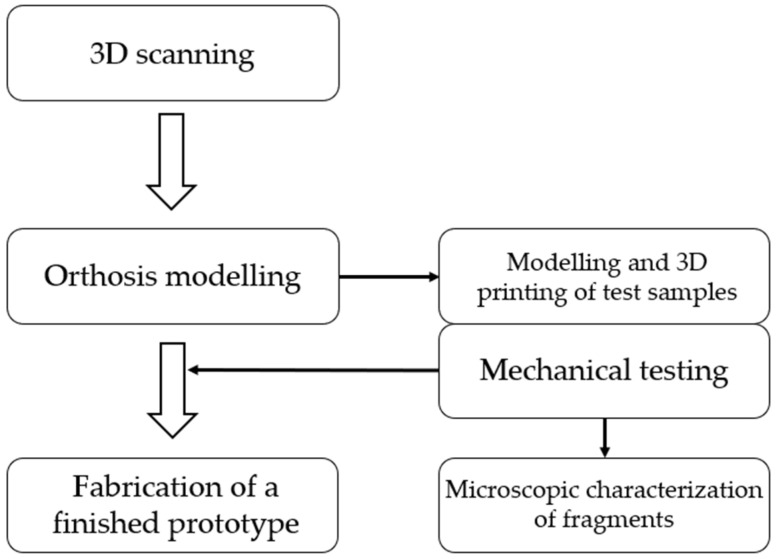
Overview of research workflow.

For the purposes of this research, a specially designed workflow, shown in Figure 1, was implemented. This workflow contains all the steps that will be further discussed.

### 2.1. 3D Scanning of a Forearm

The first step, and one of the most crucial steps in the process of fabrication of a finished prototype, was 3D scanning. This method was used for the acquisition of 3D models, and for the purposes of this research, a healthy left forearm of a 28-year-old male was scanned with the use of Vitus Smart full body 3D scanner (Human Solutions GmbH, Kaiserslautern, Germany). The scanner in question has a scanning area of 1000 by 800 mm width and depth, respectively, and a height of 2040 mm. The scanner itself has eight cameras and four lasers that are able to acquire 500,000 to 600,000 spatial points in 12 s. Unfortunately, the full body 3D scanners cannot fully encompass every point of the body, which in turn leads to occlusion of certain body regions (in this case the edge of the forearm), and this in turn results in the formation of unwanted cavities. Moreover, the acquired model has a large number of point clouds that can make the modelling process harder. To resolve the aforementioned problems, an open-source MeshLab v1.3.3. software (Istituto di Scienza e Tecnologie dell’Informazione, Pisa, Italy) was used. These goals were accomplished with the use of the Remeshing, Simplification and Reconstruction filter offered by the MeshLab. For the purposes of an anatomical model, the Poisson surface reconstruction option was used, as it allows for a fast and detailed reconstruction of a model with a high level of detail along the contours of the original model. To achieve the desired effect, a set of parameters had to be defined, and these are as follows:

- Octree depth—influences the level of detail the final model will have,
- Solver divide—influences the level a programmed conjugate gradient solver will take to solve Poisson equation,
- Samples per node—influences the minimal number of points assigned to each Octree and thus influences the level of detail,
- Surface offsetting—influences threshold correction of the surface.
- The model processing took 40 s. Aforementioned values are shown in Table 2.

**Figure 2:**
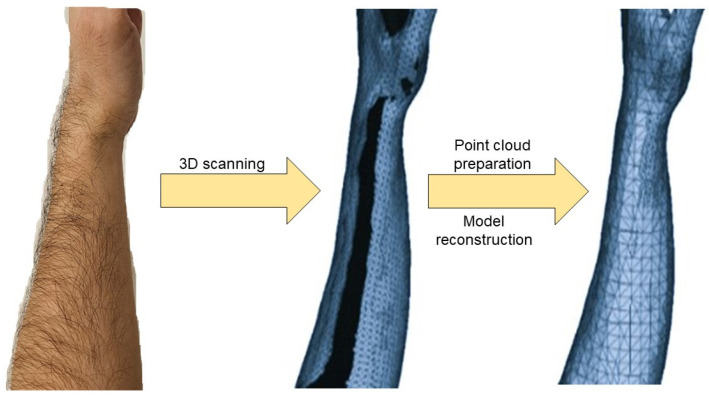
The complete workflow of the 3D scanning process. With the use of a full body 3D scanner, an initial 3D model with cavities and a large number of point clouds was acquired. Afterwards, with the use of MeshLab, a final workable model was acquired.

The complete workflow, from 3D scanning to point cloud preparation and model reconstruction, is shown in Figure 2.

### 2.2. Orthosis Modelling

The modelling was conducted with the use of the educational version of Rhinoceros 3D v.5.0. (TLM Inc., Seattle, WA, USA) in mm scale and its add-on Grasshopper in four steps:

1. 3D model segmentation,
2. Surface modelling,
3. Layout modelling,
4. Final model creation.

**Figure 3:**
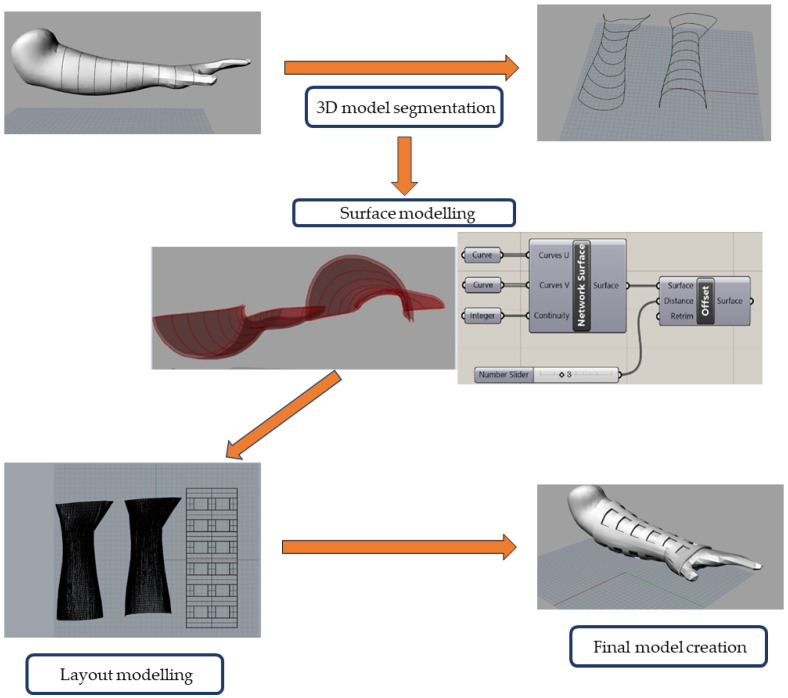
Schematic overview of the four steps used in the orthosis modelling.

The overview of the orthosis modelling is shown in Figure 3. In the first step, the 3D model was segmented into an adequate number of contour lines. This was carried out completely with the preset option *Contour*, whereupon the model was segmented with 16 rectangles, made with the option *Rectangle.* The newly made contour lines were equally spaced along the model, from the upper part of the forearm just over the tips of the fingers. For the purposes of this research, 7 out of 16 contour lines were subsequently deleted, while the remaining 9 were split in two parts, upper and lower. These contour lines enveloped the forearm model from the top part of the forearm all the way to the 2/3 of the palm. Each end of the newly made “semicircular” curves was joined and connected with the *Polyline* option. This enabled the modelling of a closed skeleton ready for the next step. It should be noted that in this step, the model can be segmented to suit the patient’s affected limb as best as possible. Therefore, the aforementioned 16 contour lines might not be suitable for shorter or longer models. It is also worth mentioning that this step is by far the longest, since it is crucial that the contour lines envelop the desired segments of the forearm. In the second step, a Rhinoceros 3D add-on, Grasshopper, was used to create surfaces along the aforementioned skeleton. This step is crucial for the designing of the shell of the final model. Therefore, a set of values was implemented. First of all, previously obtained curves were tied to the *network surface*. Precisely, two long curves in the X direction and nine short curves in the Y direction, for each skeleton, were linked to coordinates U and V, which in the case of this work can be interpreted as Y and X coordinates. Secondly, in regard to surface continuity, the *Continuity* option was tied to an integer, whose value was set as 3. *Continuity* has four different options tied to four integers, from 0 to 3, of which 3 is linked to curved surfaces. Finally, the new surfaces were offset with the *Offset* option. In this step, surfaces were offset by 3 mm in every direction so as to improve the comfort of the final object, as well as to prevent the potential occurrence of compartment syndrome and venous congestion. It should be mentioned that the template made in Grasshopper can be continuously used to offset the surface. In the third step, the 3D model that represents a flattened layout of a finished orthosis was made. The model in question was made with a ribbon-like structure. It comprises of 7 main ribbons that are connected by three vertical struts. These struts not only connect neighboring ribbons, but also enhance the overall rigidity of the final prototype. For the dimensions of the 3D layout model, it was decided that the overall thickness would be 3 mm. Further on, a 3D layout model was designed so as that every ribbon was 2 cm wide and each gap between ribbons was 2.5 cm wide (consequently, every strut had a width of 2.5 cm). The length of every ribbon was 2.2 cm. Finally, to create the final model, surfaces of upper and lower halves were transformed by the 3D layout model with the use of preset option *Flow Along Surface*, whereupon the 3D layout was bent and transformed to fit the offset surfaces made in the second step.

### 2.3. Fabrication of Test Samples

**Figure 4:**
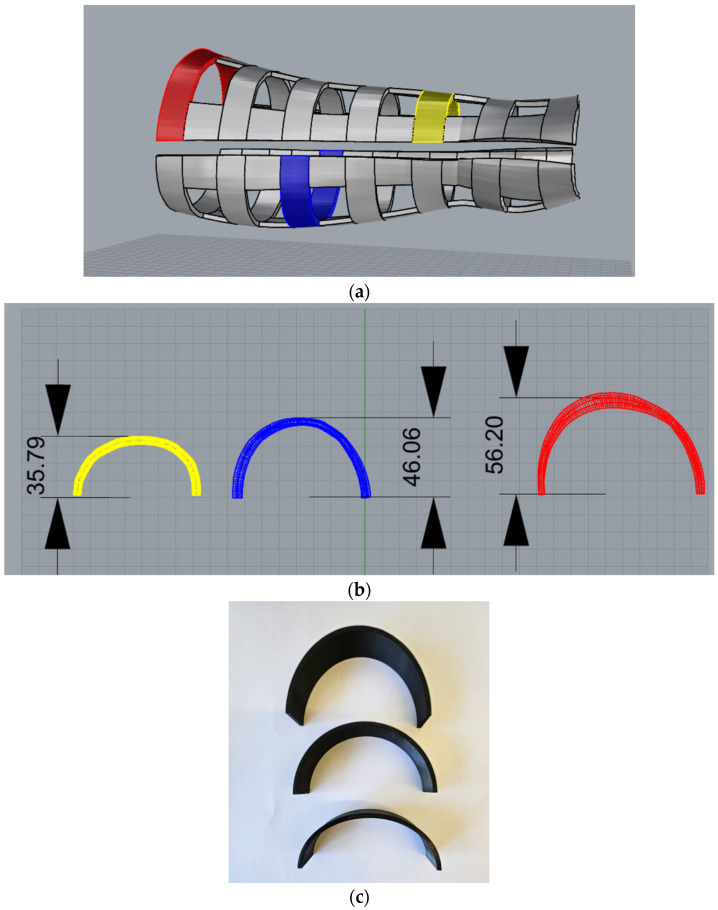
3D models of the (**a**) finished model of the orthosis with the marked ribbon segments (**b**) test samples that represent the marked ribbons; (**c**) final test samples.

With the successful outcome of the prototype orthosis modelling, mechanical properties, more specifically, the maximum load capacity, was investigated. Given that the final model was designed with the ribbon-like structure, test samples that closely represent these segments were designed using Rhinoceros, sliced with the free-to-use software PrusaSlicer v.2.6.1 (Prusa Research by Josef Prusa, Prague, Czech Republic), and fabricated with a FDM 3D printer Creality CR-10 max (Shenzen Creality3D Technology Co. Ltd., Shenzhen, China). In Figure 4, a finished model with marked ribbon segments, as well as the heights of the marked segments and the test samples that were made according to the measured heights are shown.

For the purposes of this research, an experiment that involved the investigation of the influence infill density and dimension, more specifically, test sample height, may have on the load capacity during static load was implemented. The infill density values used in this research were 20, 40, and 60%, while the heights of the test samples were set to rounded values: 56, 46, and 36 mm. These values were used due to the fact that precise heights could not be achieved with the used FDM printer. Remaining dimensions of the test samples are shown in Table 3.

Values shown in Table 3 were tailored according to the dimension of the test sample holder used in this research, and thus were not a complete representation of the aforementioned segments.

As for the 3D printing parameters, with the use of PrusaSlicer, values of the set parameters were implemented and used throughout the investigation. Values of these parameters are shown in Table 4.

These values were based on the parameters used in a previous research [48]. Furthermore, for the 3D printing, a PLA filament with black pigment (PLA Black, Azurefilm, Sežana, Slovenia) was used. The filament in question was used due to wide use of PLA, as well as the research that indicates that the addition of pigments in the polymer melt has a positive effect on the mechanical properties of 3D-printed objects [49]. Test samples were 3D printed exclusively in a vertical position, due to the 3D printer’s build volume of width, length, and height being, respectively, 400 × 400 × 470 mm. This decision was made on the basis of limitations that horizontal print may have on the outcome of the final orthosis fabrication, while acknowledging the effect this may have on the increase in general mechanical properties, as proven by Łukaszewski et al. [22].

### 2.4. Loading Capacity Test

For the purposes of the mechanical testing, the Tensolab 3000 Strength Tester (Mesdan S.p.A, Raffa, Italy) was used. Furthermore, for the purposes of this research, a flat-bottomed steel plunger with a diameter of 50 mm was used. Although this plunger is used for static puncture testing of geotextile materials, the dimensions and shape were deemed appropriate for the purposes of this research due to the fact that this investigation is focused on the determination of the maximum loading force, and orthosis could sustain along its length.

In the standard ISO 22523:2006, it is stated that for the use of external orthotic devices a “static load representing a gross single event, which can be sustained but which might render orthotic device unusable” has to be tested [24]. Since a specific mechanical testing method is not specified in the standard, it was decided that the loading capacity test, intended to represent an event in which an object of certain weight might come into contact with the orthosis and potentially compromise it, was to be investigated. Furthermore, since there are no specified parameters for the investigation, for the purposes of this research, the following parameters were set:

- Pretension—300 N;
- Speed—25 cm/min.

Pretension of 300 N was used as the minimum threshold. This value corresponds to the maximum load capacity of an eight-layer Plaster of Paris cast [17]. Therefore, if the test samples were to have lower values, they would be deemed unusable for further use. The plunger speed was used to simulate the aforementioned event, as well as cause a more violent break that could occur in real use. The load was applied on the test samples until there was a visible deformation or until a break occurred. For these purposes, mechanical testing was conducted using triplicates.

It should be noted that In their research, Górski et al. successfully investigated the mechanical properties of 3D-printed orthoses using a “quasi three-point bending test of the whole orthosis” with the minimum value threshold of 300 N, a value set in agreement with orthopedic experts [38].

### 2.5. Statistical Data Treatment

Values of loading capacity testing were analyzed with the use of two-way ANOVA and post hoc Tukey test. The relationships between independent variables and their influence on dependent variable, i.e., maximum loading force, were further explored with interaction graphs. Statistical data treatment was conducted with the use of RStudio (Posit PBC, Boston, MA, USA).

### 2.6. Microscopic Characterization of Test Sample Fragments

For the characterization of fragments that may appear after the test sample’s break due to excessive force, the portable USB digital microscope Dino-Lite PRO AM413T (Dino-Lite, Los Angeles, CA, USA), with 1.3 megapixel resolution and 60× magnification, was used. The DinoCapture 2.0. software was used to measure and take photomicrographs.

The purpose of this investigation was to assess the risk of possible skin damage that may occur in the event of orthosis break. For the purposes of this assessment, the following characteristics were taken into consideration during the microscopic characterization:

1. Occurrence of the fragments during the break;
2. Occurrence of jagged pieces along the break lines of the fragments;
3. Characteristics of jagged pieces found on the fragments.

### 2.7. Processing of the Finished Model for 3D Printing

For the purposes of finished prototype fabrication with the use of an FDM 3D printer, a finished model, obtained with the protocol described in the previous Section 2.2, first had to go through the final preparations that are intended to make the model “printable”. This is accomplished with the use of slicing software, in this case, PrusaSlicer. Given that the used test samples represented the segments of the final model, previously used parameters (Table 3) were applied again. Due to the designing process and the method of application, the orthosis consisted of two parts, upper and lower. These parts were 3D printed separately so as to avoid any possible error that may occur during the long printing process. Furthermore, due to the specific geometry of the orthosis model, supports were implemented to assure a successive printing process without possible structural collapse.

## 3. Results

### 3.1. Results of Orthosis Modelling

Orthosis modelling with the use of Rhinoceros 3D v.5.0. and its add-on Grasshopper enabled an easy and fast modelling process that was timed. The whole process took 6 min and 35 s to complete. The measured times for each step are shown in Table 5.

As seen, the second and fourth step, that is, the surface modelling and the final model creation, took the least amount of time, less than one minute, while the first and third step, the 3D model segmentation and layout modelling, took the most amount of time to complete, at more than two minutes. It should be noted that in the second step, the process will almost always take the same time, regardless of the shape and geometry of the forearm, since the Grasshopper add-on can save equations (an example can be seen in Figure 4) as templates that may be used in the future.

### 3.2. Results of Loading Capacity Testing

**Figure 5:**
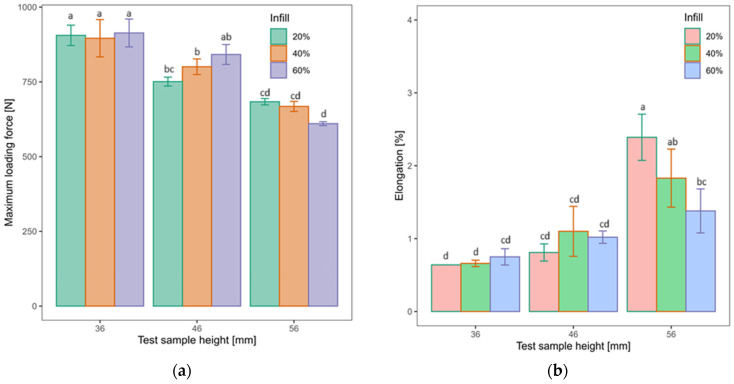
Mean values of: (**a**) maximum loading force of test samples; (**b**) elongation of test samples, with statistical analysis of presented mean values.

Results of loading capacity testing for test samples with heights 36, 46, and 56 mm are shown in Table 6 as well as Figure 5. In the table, the following symbols are used: F—maximum loading force, $\bar{X}$—arithmetic mean of maximum loading forces, ɛ—elongation at maximum loading force, $\bar{X}$ɛ—arithmetic mean of elongation, and σ—standard deviation.

Based on the obtained results, it can be surmised that the values of maximum loading force varied greatly on the basis of (i) height of test samples and (ii) infill density. Samples with 56 mm height had arguably the lowest mean values, which further decreased with the increase in infill density (684 N—20%; 668 N—40%; and 610 N—60%). On the other hand, test samples with 46 mm height had an increase at maximum force values, with the increase in infill density (751 N—20%; 801 N—40%; and 842 N—60%). Samples with 36 mm height had the highest obtained values of maximum loading force (906 N—20%; 896 N—40%; and 914 N—60%). In comparison to the values of the previous two samples, values of maximum loading force that belong to 36 mm samples did not have an increase or a decrease with the change in infill density due to the fact that four test samples with 36 mm height did not break before the plunger reached the predesigned limit. On the other hand, it can be surmised that the test sample height had a pivotal role in the elongation. The 56 mm samples had the highest observed values of elongation that, just as with maximum force before the break, decreased with the increase in infill density (2.39–20%; 1.83–40%; and 1.38–60%). The 36 mm sample had an increase in elongation with the increase in infill density, while the 46 mm sample did not have one present due to the inconsistent deviation in values of 40% infill.

To determine the influence these two factors, infill density and test sample height, may have on the values of maximum loading force, two-way ANOVA and post hoc Tukey’ HSD tests were conducted, and the obtained results are shown in Table 7.

A Two-way ANOVA revealed that there is no statistically significant influence of infill density on maximum loading force. On the other hand, a statistically significant influence of test sample height was observed. Therefore, a further post hoc Tukey test was conducted. In Table 8, results of Tukey’s HSD test are shown.

**Figure 6:**
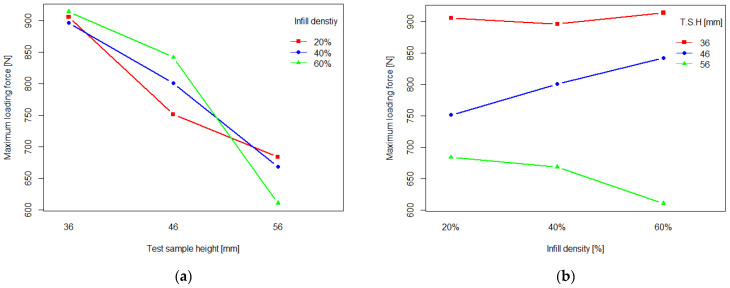
Interaction graphs representing: (**a**) interaction and influence of infill density on test sample heights’ effect on the values of loading force; (**b**) interaction and influence of test sample height on infill density’s effect on the values of loading force.

Tukey’s HSD test revealed that there was a significant difference between mean values of maximum loading force between 46 and 56 mm test samples and 36 mm samples. Finally, two-way ANOVA revealed that there was a statistically significant interaction and between infill density and test sample height’s subsequent influence on the values of loading force. To determine which of these factors had a bigger influence on the other, interaction graphs were plotted and are shown in Figure 6.

Interaction graphs show that there is an interaction between the two factors in both cases, but a clear influence of infill density on test sample height is more evident.

### 3.3. Results of Microscopic Characterization of Test Sample Fragments

Due to the brittle nature of PLA, occurrence of irregular breaks is to be expected. This may prove potentially harmful in the case of an orthotic device made from PLA breaks and splinters into sharp fragments. Therefore, microscopic characterization of fragments that occurred during the mechanical testing was conducted. To further evaluate the broken fragments, characteristics pertaining to the occurrence of fragments and jagged pieces, as well as characteristics of these pieces were observed.

The following was observed:

1. Of the 27 test samples, four of them did not break, rather they were permanently deformed.
2. Of the 23 test samples that did break into fragments, three of them broke off into more than two fragments—three, to be more precise.
3. Of the 23 test samples that were fragmented, 17 test samples had jagged pieces along the break lines.
4. Of the 17 fragments, five of them that came from the same test sample, did not have jagged pieces on all fragments.

The lengths of these jagged pieces were measured using the DinoCapture’s measuring option and recorded in Table 9.

**Figure 7:**
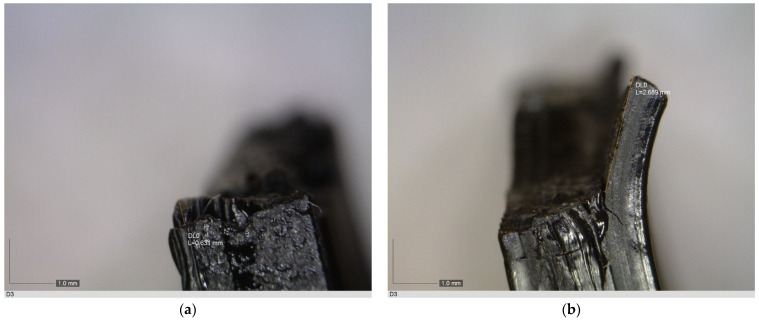

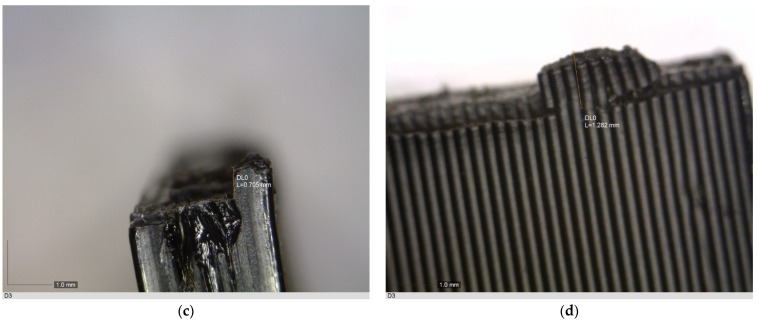
Photomicrographs of fragments with: (**a**) a small chipped jagged piece; (**b**) a single pointed sharp tip; (**c**) a small flat jagged piece; (**d**) a small flat tip.

The obtained values, more precisely the maximum and minimum length, as well as average and median length, indicate that on average, jagged pieces were not longer than 1 mm, while the maximum measured length was not longer than 3 mm. Photomicrographs of some of the fragments are shown in Figure 7.

With the use of microscopic characterization, it has been observed that on average, the jagged pieces found on the fragments had a flat-chipped appearance, as seen in Figure 7a,c,d, while very few of them had a single pointed sharp tip, such as a Figure 7b.

### 3.4. Results of Finsihed Model 3D Printing

**Figure 8:**
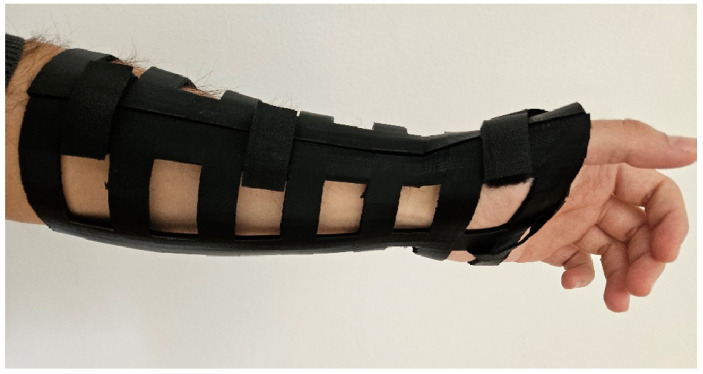
3D-printed orthosis placed on the forearm of the participant.

According to the results obtained from the mechanical testing, 3D printing of the finished model was conducted with a 40% infill density. The process took 18 h and 42 min; more precisely, 9 h and 11 min for the upper part, and 9 h and 33 min for the lower part. As for the support type, organic type was used due to the ease of removal. Organic type was the best choice due to the fact that after the printing had finished, supports were removed easily by hand without the use of any tools, which is not possible with other support types (grid and snug) that fully envelop the model. Afterwards, the finished prototype halves were easily joined together using Velcro straps. For this process, two people were involved, the test subject on whose forearm the orthosis was placed and another person who in this case took the role of the medical personnel in charge of orthosis application. Velcro straps were chosen since they would not interfere with the clothes. The finished model, placed on the forearm of the participant, is shown in Figure 8.

## 4. Discussion

In this research, a great deal of focus was placed on the development of an easy-to-use template using a 3D modelling software, Rhinoceros, and its built-in add-on, Grasshopper. Based on the obtained results for the overall 3D modelling workflow, the whole process lasted 6 min and 35 s, which, coupled with the 62 s 3D scanning and postprocessing workflow, made the whole process last less than 10 min (7 min and 37 s). Furthermore, when it comes to the structure of the orthosis, ribbon-like shape was chosen due to its simplicity. This decision enabled the modelling of a device without any special training or education, as well as adequate 3D printing time, which is imperative for real-time use in the medical field. To further evaluate the potential real-time use, an investigation of using the procedure described here, tailored towards individual proportions, should be conducted on a larger population.

The loading capacity tests conducted on the test samples, which represent segments of the finished orthosis, indicated that in comparison to the conventional Paris of Plaster casts, 3D-printed 3 mm samples have at least more than double, and at most cases, more than triple the loading capacity of Paris of Plaster casts. Furthermore, the mechanical testing indicated that the maximum loading forces of the test samples varied greatly. With the use of two-way ANOVA and post hoc Tukey’s HSD test, it was determined that (i) infill density did not have a significant influence on the maximum loading force values; and (ii) test sample height had a significant influence on the obtained values. Moreover, it was observed that mean values of test samples with 36 mm height were significantly greater than those of 46 mm, especially 56 mm. Finally, it was observed that there was a significant interaction between infill density and height of the test samples. With the use of interaction graphs, it was determined that when it comes to the interaction of the two factors and their impact on the maximum loading force, infill density had a much greater influence on the height of the test samples than the other way around. Furthermore, the interaction graph shown in Figure 6a indicates that when it comes to the choice of the infill density, 40% infill is the best option due to an almost linear decrease in maximum loading force with the increase in the test sample height, as well as a decrease in samples with other infill densities lacked. Based on this conclusion, it was decided that the 40% infill was the most appropriate infill density for latter stages of 3D printing. As for the decrease in the maximum loading force with the increase in the test sample height, as seen in Figure 6a, it was observed that due to the nature of the flat-bottomed plunger that was used in this investigation, the distribution of force along the test sample surface, as well as the ability to deform, was not equal among the samples and infill densities of the same amount. The flatter 36 mm test samples were able to distribute the incoming force on the larger surface in comparison to the “spikier” 56 mm samples. When it comes to the 56 mm samples, it was observed that with the increase in the infill density, the deformation of the test samples decreased, which the values of elongation at maximum loading force have shown. On the other hand, 46 mm samples had an almost linear increase in the values of maximum loading force with the increase in the infill density. They were able to distribute the incoming force more evenly. Moreover, unlike with the 56 mm samples, their ability to deform increased with the increase in the infill density, which was corroborated by the values of elongation at maximum loading force. To fully evaluate the mechanical properties of the model used in this investigation, a detailed FEA analysis, as Łukaszewski et al. conducted for their orthosis model, should be conducted [22].

With the use of the microscopic characterization, it was observed that the majority of the samples, 85%, had fragmented due to an excessive force. Of these samples, 74% had jagged pieces along the break lines. The pieces in question were found to have mostly a flat chipped appearance, while the few of them had a pointed sharp tip. As for the lengths of these pieces, it was observed that the length varied between 0.270 and 2.689 mm, with the average and median lengths being close (average—0.996 mm, median—0.834 mm). Although not particularly long, jagged pieces that were found could still pose a piercing risk since their length is well over the thickness of the epidermis [50], and thus a substantial damage of the skin surface could potentially occur. With the revelation of the maximum loading forces and their variation along the orthosis as a whole, a weight of approximately 68 kg (668 N equates approximately to this weight) could compromise the orthosis. This type of investigation has not been conducted in previous research pertaining to the development of the orthotic devices [21,22,44,45] and should be crucial when it comes to the decisions regarding the choice of building polymer, as all potential risk factors should be assessed. Therefore, a more detailed investigation into not only potential damage, but also key parameters, such as the pressure needed for the puncture, along the line of the investigation Shergold and Fleck [51] conducted, should be conducted.

As previously stated, the model of the orthotic device, designed for this investigation, was made with the ease of modelling in mind. Furthermore, it was observed that the implementation of the simple ribbon-like geometry of the orthosis had a direct influence on the printing time. Based on the previously established results of the mechanical testing, an orthosis with 40% infill was 3D printed in 18 h and 42 min, which is less time than it took to 3D print an orthosis with a more complicated design geometry [21].

## 5. Conclusions

As presented in this research, an orthosis intended for DRF treatment was successfully designed and fabricated according to the obtained forearm 3D scan. With the use of 3D technologies, a developed workflow could potentially be used in real-time application due to its simplicity. Further on, the following was concluded:

- PLA 3D-printed orthosis can withstand forces that would compromise conventional Paris of Plaster casts.
- Developed workflow for 3D scanning, modelling, and printing took less than 24 h to complete.
- Due to excessive forces during the break, resulting fragments and jagged pieces could potentially damage human skin.

In the end, it is worth noting that this investigation should be considered a preliminary investigation towards the real-time application of a simplistic model presented in this research. Therefore, further investigations are warranted, especially when it comes to the potential risk fragments may cause.

## Figures and Tables

**Table 1:** Advantages and disadvantages of various thermoplastic polymers and composites used in orthopedics.

Material Researched	Advantages	Disadvantages
PCL	Great dimensional accuracy and stability of the finished orthosis [23].	Long calibration needed to achieve the optimal production [23].
ABS	High impact resistance [38]; ideal material for supportive structural parts [38].	Not suitable for direct contact with skin; Negative impact on the environment [38].
Polyethylene terephthalate glycol	Ideal material for supportive structural parts [39]; great elasticity and impact behaviour [40].	High cost of filament. Almost the double than that of PLA [40]
Nylon	Great bending strength and impact scratch resistance [38].	High cost of filament and problems with printing [38].
Thermoplastic Polyurethane	Desirable elasticity for padding and strapping [39].	High cost and difficulty of the printing process [39].
Polypropylene	Desirable elasticity for padding and strapping [39].	Polypropylene is expensive and difficult to print with [39].
Polyether ether ketone	High crystallinity over a large temperature range—great mechanical properties and biocompatibility [41].	High melting temperature during printing can induce deformations [41]; problematic for use with conventional material extrusion 3D printers [42].
ABS/ short glass fibre composite	Increased mechanical properties in comparison to pure ABS [43].	Not suitable for direct contact with skin. Negative impact on the environment [36].

**Table 2:** Set parameters used for the successful point cloud preparation and model reconstruction, i.e., the decrease in point clouds and removal of cavities that occur due to the occlusion.

Octree Depth	Solver Divide	Samples Per Node	Surface Offsetting
6	6	1	1

**Table 3:** Values of test samples inner and outer diameter, width, and depth.

Inner Diameter [mm]	Outer Diameter [mm]	Width [mm]	Depth [mm]
86	92	3	20

**Table 4:** Set parameters used in 3D printing of the test samples.

Layer Height [mm]	Fill Angle [°]	Print Speed [mm/s]	Extrusion Temperature [°C]	Bed Temperature [°C]	Inner Infill Pattern	Outer Infill Pattern
0.3	45	60	215	60	Stars	Rectilinear

**Table 5:** Measured time of four modelling steps: segmentation of a forearm model; surface modelling; layout modelling; and final model creation.

Step	1st Step	2nd Step	3rd Step	4th Step
**Time [s]**	169	45	131	36

**Table 6:** Results of loading capacity test of samples. Values of maximum loading force and elongation present at maximum loading force are shown.

Infill Density [%]	Test Sample Height [mm]	F [N]	$\bar{X}$ [N]	σ [N]	ɛ [%]	$\bar{X}$	σ [%]
20	36	886	906	34.064	0.64	0.64	0
		886			0.64		
		945			0.64		
40	36	857	896	62.426	0.63	0.66	0.044
		968			0.71		
		863			0.64		
60	36	967	914	46.576	0.78	0.75	0.112
		881			0.63		
		893			0.85		
20	46	768	751	14.933	0.94	0.81	0.118
		745			0.78		
		740			0.71		
40	46	804	801	26.160	0.94	1.10	0.343
		773			0.86		
		825			1.49		
60	46	877	842	33.247	1.11	1.02	0.085
		811			1.02		
		837			0.94		
20	56	695	684	14.933	2.43	2.39	0.317
		682			2.05		
		674			2.68		
40	56	662	668	26.160	1.60	1.83	0.398
		687			2.29		
		655			1.60		
60	56	617	610	33.247	1.30	1.38	0.302
		604			1.71		
		610			1.12		

**Table 7:** Results of two-way ANOVA test.

Factor	Df	Sum Sq	Mean Sq	F-Value	*p*-Value
Infill dens.	2	412	206	0.191	0.82745
T.S. height	2	28,574	142,874	132.879	1.66 × 10^−11^ ***
Inf. dens. and T.S. height	4	2137	5344	4.970	0.00707 **
Residuals	18	193,554	1075		

Where: *** *p*-Values are extremely significant; ** *p*-Values are very significant.

**Table 8:** Results of Tukey’s HSD test.

Compared Heights	Mean Value Diff.	Conf. Int. Lower Bound	Conf. Int. Upper Bound	*p*-Value
46–36	−107.3333	−146.7837	−67.88294	5 × 10^−6^
56–36	−251.1111	−290.5615	−211.66072	0
56–46	−143.7778	−183.2282	−104.32739	1 × 10^−7^

**Table 9:** Measured lengths of jagged pieces found on 17 fragments.

Test Sample	Length of the Jagged Piece [mm]
1	0.631
2	1.225
	0.503
3	0.513
4	0.800
	0.700
5	0.808
6	1.478
	1.590
7	0.825
	0.908
8	0.830
	1.013
9	1.713
	1.954
10	1.579
	1.282
11	1.456
	2.689
12	0.905
13	0.705
	0.796
14	0.369
	0.415
15	0.837
	1.095
	0.988
16	0.727
17	0.281
	0.270
Average length [mm]	0.996
Median length [mm]	0.834
Maximum length [mm]	2.689
Minimum length [mm]	0.270

## Data Availability

Data underpinning this work will be available upon request.
